# Adverse Childhood Experiences and Police Contact in Canada

**DOI:** 10.1177/08862605241270047

**Published:** 2024-08-14

**Authors:** Alexander Testa, Benjamin Jacobs, Jennifer Thompson, Nelson Pang, Dylan B. Jackson, Jason M. Nagata, Kyle T. Ganson

**Affiliations:** 1 University of Texas Health Science Center at Houston, USA; 2 Duke University Medical Center, Durham, NC, USA; 3 University of Toronto, ON, Canada; 4 Johns Hopkins Bloomberg School of Public Health, Baltimore, MD, USA; 5 University of California, San Francisco, USA

**Keywords:** physical abuse, child abuse, neglect, child abuse, criminology

## Abstract

A growing body of research has demonstrated that adverse childhood experiences (ACEs) are a risk factor for criminal justice system contact. However, much of this research is limited by (1) being conducted in the United States and (2) a lack of details on specific types of harmful experiences of criminal justice system contact, such as police contact characterized by intrusion or harassment. Using survey data from 940 individuals aged 16 to 30 in Canada from the Canadian Study of Adolescent Health Behaviors, this study investigates the relationship between ACEs and police contact, focusing on encounters involving intrusion or harassment. Results from logistic and multinomial logistic regression analyses reveal that individuals with high ACE exposure, particularly those with four or more ACEs, are more likely to have police contact, including experiences of intrusion and harassment. The results are significant in understanding the interplay between childhood trauma and later encounters with the criminal justice system, emphasizing the need for trauma-informed approaches in policing and healthcare. The study highlights the importance of early interventions to mitigate the effects of ACEs and prevent adverse outcomes in police interactions.

## Introduction

Adverse childhood experiences (ACEs) encompass a spectrum of traumatic events that transpire during formative developmental years (i.e., before 18 years old), including but not limited to abuse, neglect, and household dysfunction (Felitti et al., 1998; Sacks & Murphey, 2018). The detrimental effects of ACEs are profound, impacting health and developmental outcomes across the lifespan (Bellis et al., 2019; Hughes et al., 2017; Kalmakis & Chandler, 2015). Recent data underscores the pervasive nature of ACEs, indicating that approximately three in five Canadian adults aged 45 to 85 years old have experienced at least one ACE (Joshi et al., 2021).

While ACEs are associated with several negative consequences over the life course, the extent to which ACEs are linked to distressing encounters with law enforcement remains insufficiently explored, particularly within the Canadian context. Adverse experiences during childhood and adolescence have detrimental impacts on behavioral health and development and can contribute to later conduct and interpersonal difficulties (Austin, 2018; Campbell et al., 2016). These difficulties often manifest as substance use, school truancy, and/or externalizing behaviors, which further increase the chances of police contact (Afifi et al., 2020; Baiden et al., 2020; Bevilacqua et al., 2021; Boccio et al., 2025; Henry et al., 2021; Hunt et al., 2017; Jackson et al., 2022; Poole et al., 2017). Several cross-sectional studies have previously reported significant correlations between experiencing ACEs and an increased likelihood of individuals coming into contact with the criminal justice system, including arrest and incarceration (Graf et al., 2021; Stinson et al., 2016). More recently, longitudinal research has provided additional evidence of a positive temporal relationship whereby those with early ACE exposures also report increased subsequent police contact (Jackson et al., 2022; Testa & Jackson, 2022; Testa et al., 2022a).

Notwithstanding the knowledge gained from this extant research regarding ACEs and criminal justice contact, several critical gaps exist. First, studies on ACEs and police contact have been almost exclusively conducted in the United States (Graf et al., 2021; Stinson et al., 2016; Testa & Jackson, 2022; Testa et al., 2022), with one study from the United Kingdom (Jackson et al., 2022). Expanding the knowledge of this relationship to contexts outside of the United States to Canada is pivotal due to the country’s unique social policies, healthcare system, and cultural mosaic, which may contribute to differing patterns of ACEs and subsequent interactions with law enforcement compared to the United States (Thomas & Sands, 2023). Additionally, differences in policing strategies and criminal justice policies between Canada and the United States could influence the nature and frequency of police contact among individuals with ACEs (Ben-Porat, 2008). Investigating the relationship between ACEs and police encounters across diverse national contexts is crucial to understanding the unique mechanisms that ACEs serve as a risk factor for criminal justice system contact across countries with different cultures and criminal justice systems, which will help to develop cross-cultural strategies for prevention and intervention.

Second, there is an insufficient understanding of whether ACEs differentially impact police stop incidents that encompass encounters involving verbal or physical intrusion or harassment predicated on a person’s physical appearance, identity, or demographic profile. Indeed, prior research illustrates that individuals with greater ACE exposure are more likely to experience police stops (Jackson et al., 2022), arrests (Graf et al., 2021; Stinson et al., 2016; Testa et al., 2022), and unfair police treatment (i.e., unfairly stopped, searched, or questioned by the police) (Testa & Jackson, 2022). However, understanding the relationship between ACEs and specific adverse experiences with police contact rooted in diverse forms of unjust intrusion or harassment is critical because these forms of police interactions can severely harm one’s health and development over the life course (Baćak & Apel, 2020; Dennison & Finkeldey, 2021; Geller et al., 2014; Jackson et al., 2020; McFarland et al., 2018; Testa et al., 2022b; Turney, 2021).

This paper endeavors to fill the research mentioned above gaps by examining the relationship between ACEs and police contact within the Canadian context. Specifically, drawing on survey data from adolescents and young adults in Canada, the current study assesses the relationship between ACEs and encounters with the police, including instances involving intrusion or harassment.

## Methods

The data used in this study were obtained from the Canadian Study of Adolescent Health Behaviors (*N* = 940; ages 16–30). The recruitment of the initial sample took place in November and December 2021 through Instagram and Snapchat advertisements, and a second round of data collection occurred in November and December 2022 by contacting original participants via email to participate in a follow-up survey. The data collection for this study was conducted online via Qualtrics. Participants were given the opportunity to enter a drawing for one of three Apple iPads as an incentive for their participation. Multiple techniques were employed to ensure data quality, including reCAPTCHA verification for the survey, attention checks, and honeypot items (Xu et al., 2022). Features on Qualtrics were also enabled to prevent participants from taking the survey multiple times and to prevent the survey from appearing on search engine searches. Ethical approval was obtained from the research ethics board at the University of Toronto (#41707), and written informed consent (via checkbox) was obtained from all participants.

### Measures

#### Dependent Variables

Three dependent variables were measured in the follow-up survey, assessing the experiences of participants with police contact at any point prior to their completion of the study survey.

*Police contact* is a binary variable based on a question asking participants, “Have you ever been stopped, searched, or questioned by the police?” (1 = *yes*; 0 = *no*).

*Police contact with intrusion* is based on a question asking, “Now think about the time you were stopped by the police that stands out most in your mind. During this encounter, did the officer(s) [select all that apply]”: (a) use harsh language, (b) frisk or search you, (c) threaten physical force, (d) use physical force, (e) arrest you, or (f) other [respondent textual description of unjust police behavior]? (0 = *never stopped by police*; 1 = *stopped without intrusion* [stopped by police and responded “no” to items a–f], and 2 = *stopped with intrusion* [stopped by police and responded “yes” to at least one item a–f]) (Alang et al., 2021).

*Police contact with harassment* is based on a question asking participants, “Have you ever felt you were harassed or made to feel inferior for any reason by the police [select all that apply]”: (a) no, (b) yes, based on my race, ethnicity, or skin color, (c) yes, based on my language or accent, (d) yes, based on my immigration status, (e) yes, based on my sexual orientation, (f) yes, based on my gender identity, (g) yes, based on my sex, (h) or yes, based on my physical appearance, or (i) other [i.e., age; disability]) (0 = *never stopped by police*; 1 = *stopped without harassment* [stopped by police and responded “no” to items a–i], and 2 = *stopped with harassment* [stopped by police and responded “yes” to at least one item a–i] (Alang et al., 2021).

#### Independent Variables

ACEs were assessed at the follow-up survey and measured participants’ experiences prior to 18 years of age. ACEs included abuse (physical, emotional, and sexual), household dysfunction (parental divorce, family violence, presence of substance abuse, family mental health challenges, suicide, or incarcerated member of the household), and neglect (emotional and physical). Each question had the response option of “no” or “yes” with yes responses indicating the presence of each ACE. The ACEs items were adapted from prior research (Felitti et al., 2019). These 15 binary measures were summed into a cumulative ACEs score (0–15), which was then used to create a categorical count of ACEs, including 0, 1, 2, 3, 4, or more ACEs. The specific ACE, including definition and participant prevalence, is listed in Supplemental Appendix A.

#### Control Variables

Sociodemographic variables included self-reported age, gender (cisgender woman, cisgender man; due to the small sample sizes, transgender and gender-expansive participants were collapsed into a single group), race/ethnicity (collapsed to White or non-White due to limited racial variation in the sample), sexual orientation (heterosexual, gay/lesbian, bisexual, or questioning, queer, other), relationship status (in a relationship or not in a relationship), personal income ($0–$24,999, $25–$49,999, $50,000–$74,999, or ≥ $75,000), and highest competed education (high school diploma or less, college degree, master’s degree or higher). Supplemental models include additional control variables for *involvement in violence* and *illicit drug use* in the previous 12 months.

### Statistical Analysis

The relationship between ACEs and any police contact is assessed with multiple logistic regression. Multinomial logistic regression is used to assess the relationship between ACEs and (a) police stops with intrusion and (b) police stops with harassment, adjusting for control variables. We then perform two supplemental analyses: (1) assess the relationship between each specific ACE and the outcome variables, and (b) repeat the main analyses accounting for patterns of involvement in violent crime and illicit drug use. Across all analyses, multiple imputations with chained analysis were performed in Stata 17 (Stata Copr) to address missing data, resulting in 20 multiply imputed datasets. Statistical significance was determined using two-sided *p* < .05. All analyses were conducted using Stata 17.

## Results

### Sample Overview

Table 1 provides the summary statistics of the analytic sample. Over 1 in 5 (21.7%) respondents reported zero ACEs, 14.6% reported 1 ACE, 11.8% reported 2 ACEs, 11.3% reported 3 ACEs, and 40.7% reported 4 or more ACEs. Overall, 23.2% of participants reported experiencing any police contact, 10.8% reported police contact without intrusion, 12.3% reported police contact with intrusion, 14.5% reported police contact without harassment, and 8.6% reported police contact with harassment.

**Table 1.** Demographic Characteristics of Participants from the Canadian Study of Adolescent Health Behaviors (*N* = 940).

Variable	% Or mean
Police contact	
Any police contact	23.2
Police contact with intrusion	
Never stopped	76.8
Stopped without intrusion	10.8
Stopped with intrusion	12.3
Police stop with harassment	
Never stopped	76.8
Stopped without harassment	14.5
Stopped with harassment	8.6
Number of ACEs	
0	21.7
1	14.6
2	11.8
3	11.3
4+	40.7
Age [*M* (*SD*)]	23.3 (4.5)
Race/ethnicity	
White	62.0
Non-White	38.0
Gender	
Cisgender woman	57.0
Cisgender man	33.5
Transgender/gender expansive	9.5
Sexual orientation	
Heterosexual	54.5
Gay/lesbian	9.4
Bisexual	17.6
Question, questioning, other	18.4
Relationship	
Not in a relationship	47.9
In a relationship	52.1
Personal income	
$0 to $24,999	46.8
$25,000 to $49,999	19.5
$50,000 to $74,999	16.9
≥$75,000	16.7
Highest completed education	
High school diploma or less	33.6
Undergraduate/college	48.8
Masters or higher	17.7
Illicit drug use	27.8
Involvement in violent crime	5.1

*Note.* ACEs = adverse childhood experience; *M* = mean; *SD* = standard deviation.

### ACEs and Police Contact

Table 2 provides the multiple logistic regression of the number of ACEs and experiencing any police contact. After adjusting for age, race/ethnicity, gender, sexual identity, income, and the highest level of education completed, respondents with four or more ACEs have a 77% higher odds of police contact relative to respondents with no ACEs (OR = 1.77, 95% CI [1.13, 2.78]). The multinomial logistic regression results in Table 3 show that relative to the reference group of respondents with no ACEs and the base outcome of no police contact, having four or more ACEs is associated with a 108% higher risk of police contact with intrusion (relative risk ratios [RRR] = 2.08 [1.17, 3.71]) and 325% higher risk of police contact with harassment (RRR = 4.25 [1.83, 9.86]), even after adjusting for age, race/ethnicity, gender, sexual identity, income, and highest level of education completed.

**Table 2.** Associations Between the Number of ACEs and Any Police Contact Among Participants in the Canadian Study of Adolescent Health Behaviors (*N* = 940).

Variables	AOR [95% CI]^a^	*p*
Number of ACEs		
0	Ref.	Ref.
1	1.14 [0.64, 2.01]	.655
2	0.86 [0.44, 1.67]	.652
3	1.05 [0.56, 1.99]	.871
4+	**1.77 [1.13, 2.78]**	**.013**

*Note.* Values in bold are significant with *p* < .05. ACEs = adverse childhood experience; AOR = adjusted odds ratio.

a Analyzes adjusted for age, race/ethnicity, gender, sexual identity, income, and highest level of education completed.

**Table 3.** Multinomial Logistic Regression of Relationship Between Number of ACEs and Police Stops with Intrusion and Police Stops with Harassment Among Participants in the Canadian Study of Adolescent Health Behaviors (*N* = 940).

Type of Police Contact	Police Stop Intrusion				Police Stop Harassment			
	Never Stopped vs. Stopped w/o Intrusion		Never Stopped vs. Stopped w/ Intrusion		Never Stopped vs. Stopped w/o Harassment		Never Stopped vs. Stopped w/ Harassment	
	RRR [95% CI]^a^	*p*	RRR [95% CI]^a^	*p*	RRR [95% CI]^a^	*p*	RRR [95% CI]^a^	*p*
Total number of ACEs								
0	Ref.	Ref.	Ref.	Ref.	Ref.	Ref.	Ref.	Ref.
1	1.30 [0.62, 2.76]	.488	0.96 [0.45, 2.07]	.928	1.18 [0.63, 2.19]	.601	1.10 [0.35, 3.44]	.872
2	0.82 [0.31, 2.11]	.669	0.89 [0.39, 2.05]	.784	0.76 [0.35, 1.66]	.492	1.33 [0.42, 4.21]	.622
3	1.10 [0.46, 2.63]	.871	1.00 [0.44, 2.28]	.996	0.90 [0.43, 1.88]	.778	1.74 [0.57, 5.28]	.327
4+	1.48 [.78, 2.81]	.299	**2.08 [1.17, 3.71]**	**.013**	1.15 [0.67, 1.96]	.609	**4.25 [1.83, 9.86]**	**.001**

*Note.* Values in bold are statistically significant at *p* < .05. ACEs = adverse childhood experience; RRR = relative risk ratios; CI = confidence interval.

a Analyses adjusted for age, race/ethnicity, gender, sexual identity, and highest level of education completed.

### Supplementary Analysis

We conducted supplementary analyses of the models in Tables 2 and 3 by expanding control variables to include involvement in violence and illicit drug use in the past 12 months. Supplemental Appendix B shows that even after controlling for violence and illicit drug use, in addition to the previous control variables, a positive association remains between four or more ACEs and any police contact. However, the relationship is partially attenuated (OR = 1.59, 95% CI [1.00, 2.52]). Supplemental Appendix C shows that while controlling for these variables, a relationship remains between four or more ACEs and having reported police stop with intrusion (RRR = 1.86 [1.04, 3.35]) and police stop with harassment (RRR = 3.71 [1.58, 8.68]) compared to those reporting no police contact and no ACEs. Supplemental Appendices D and E replicate the main analyses, examining the relationship of each specific ACE item on any police contact, police contact with intrusion, and police contact with harassment. Appendices F to I reproduce the results using a top-coding of ACEs at ≥5 or ≥6 ACEs. Finally, Supplemental Appendix J plots the predicted probabilities of police contact by a discrete count of ACEs.

## Discussion

### Summary of Findings

Using a sample of adolescents and young adults from Canada, the current study finds that high ACE exposure—especially four or more ACEs—was associated with a greater likelihood of having any police contact and a greater risk for negative interactions with the police, including police contact with intrusion and police contact with harassment. Moreover, these results remain after adjusting for race/ethnicity, gender, sexual identity, income, and education levels, and persist even when accounting for previous patterns of involvement in violent crime and substance use. To our knowledge, this study is the first to show a relationship between ACEs and police contact in Canada and corroborates prior literature using samples from other countries, such as the United States (Fleming & Nurius, 2020; Graf et al., 2021; Stinson et al., 2016; Testa & Jackson, 2022; Testa et al., 2022a; Zettler et al., 2018) and the United Kingdom (Jackson et al., 2022).

Participants in this study were particularly diverse in terms of their gender, with 9.5% of participants having identified as transgender or gender expansive and sexual minority orientation, with 36% who identified as bisexual, queer, questioning, or other (17.6% bisexual; 18.4% queer, questioning, or other). Previous studies have highlighted a significant increase in the odds of police contact for persons of gender and sexual minority status compared to heterosexual individuals in Canada (Testa et al., 2024). Future work studying the relationships between ACEs and police contact in Canada and, more broadly, should seek to collect more nuanced data on gender and sexual minority membership, especially given findings indicating that police stops perceived as unfair occur more frequently for sexual minority women than sexual minority men (Baćak et al., 2021).

ACEs, encompassing a range of traumatic childhood experiences, have long been associated with adverse health outcomes (Bellis et al., 2019; Felitti et al., 1998; Hughes et al., 2017; Kalmakis & Chandler, 2015). The elevated likelihood of police contact with intrusion or harassment among those with high ACE exposure underscores the interconnectedness of early life traumatic experiences and subsequent contact with the criminal justice system (Graf et al., 2021). The heightened risk of police interactions observed in this study among those with high ACE exposure raises concerns about the potential retraumatization of individuals who have experienced significant early adversity, especially considering the link between adverse experiences with police contact and post-traumatic stress responses (Dennison & Finkeldey, 2021; DeVylder et al., 2020; Gearhart et al., 2022a, 2022b, 2023; Jackson, 2021; Jackson et al., 2019; Turney, 2021).

### Policy Implications

These results also have significant implications for the Canadian criminal justice system. The increased likelihood of police contact with intrusion or harassment among young adults with four or more ACEs suggests that the system may inadvertently exacerbate the vulnerabilities of this already marginalized population that has endured a great deal of trauma. Policymakers and criminal justice professionals should consider trauma-informed approaches that address the history of trauma and adversity experienced by individuals (Bateson et al., 2020; Gill et al., 2016). Hosting seminars and training sessions led by trauma-informed professional counselors or public health experts can teach police and policymakers alike to serve and view the world through an ACEs lens. In practice, using trauma-informed language can foster connection and alleviate the traumatic victimization these individuals have endured throughout their interaction with the justice system (Bateson et al., 2020). In addition, the implementation of de-escalation training for police officers is a promising avenue to reduce the prevalence of negative police-citizen encounters. Such training can provide officers with essential skills in communication, empathy, and conflict resolution, potentially decreasing the incidence of violent confrontations and improving overall public safety (Bennell et al., 2020; Engel et al., 2020). The findings also underscore the importance of interdisciplinary collaboration between public health and criminal justice sectors to develop policies and practices prioritizing the well-being and rehabilitation of individuals with ACEs, ultimately contributing to a more equitable criminal justice system (Jackson, 2021).

Furthermore, as the Canadian health system strives to address health inequities and promote a holistic approach to well-being, these findings underscore the need for targeted interventions and support systems that recognize the complex interplay between childhood trauma, mental health, and interactions with the criminal justice system. In particular, healthcare providers play a pivotal role in identifying and mitigating the impacts of these intertwined challenges to promote resilience and well-being among individuals with high ACE exposure (Barnes et al., 2020; Rariden et al., 2021). Screening tools and strategies can be instrumental in this endeavor, helping providers identify at-risk persons and tailor interventions accordingly (Chandler et al., 2015; Lanier et al., 2018). By systematically assessing ACEs and police contact experiences, healthcare professionals can better understand patients’ unique needs and offer targeted services such as trauma-informed care, mental health support, and community resources essential for mitigating trauma and fostering healing (Dube, 2018; Oral et al., 2016). This holistic approach underscores the vital role healthcare providers can play in breaking the cycle of trauma and supporting individuals on their path to improved health (Kalmakis & Chandler, 2015; Kia-Keating et al., 2019).

### Limitations

The current study has limitations that offer opportunities for further exploration in future research. First, the data were collected using non-random probability sampling methods and, as such, did not represent the entire Canadian population. While there is demographic diversity, and the sample included participants from all Canadian provinces and territories except Prince Edward Island, it is important to note that the online recruitment strategy may have introduced selection bias if there was a disproportionate response by those with higher ACE exposures. Second, the data originated from a survey inquiring about police contact and ACEs, which relied on retrospective self-reported measures, potentially introducing reporting biases. Nonetheless, self-reports remain the prevailing method for gathering information on both experiences with police contact and ACEs. Third, the data are cross-sectional; therefore, we cannot determine the timing of police contact in individuals’ life courses or evaluate patterns of recurrent police interactions over time. To address these limitations, a significant avenue for future research in Canada would involve addressing the questions raised in this study using nationally representative data drawn from probability sampling methods, making findings more generalizable. Finally, work that includes the acquisition of longitudinal data on ACEs and police contact, especially from more racially diverse samples, would be worthwhile.

## Conclusions

Using a recent national sample of adolescents and young adults from Canada, this study found a positive relationship between the accumulation of ACEs and experiences with police contact, including police contact with intrusion and police contact with harassment. These findings suggest that high ACE exposure—particularly four or more ACEs—serves as a risk factor for intrusive experiences with police. Given that both ACEs and negative police contact have wide-ranging consequences for health over the life course, these findings underscore the critical need for early interventions to mitigate the negative effects of ACE exposure.

## Supplemental Material

sj-docx-1-jiv-10.1177_08862605241270047 – Supplemental material for Adverse Childhood Experiences and Police Contact in Canada Supplemental material, sj-docx-1-jiv-10.1177_08862605241270047 for Adverse Childhood Experiences and Police Contact in Canada by Alexander Testa, Benjamin Jacobs, Jennifer Thompson, Nelson Pang, Dylan B. Jackson, Jason M. Nagata and Kyle T. Ganson in Journal of Interpersonal Violence
