# Supplementary material for: Adverse Childhood Experiences and Police Contact in Canada
Source: J Interpers Violence. 2024 Aug 14;40(9-10):2188–204. doi: 10.1177/08862605241270047 (PMC11951447; doi:10.1177/08862605241270047)
Supplement: sj-docx-1-jiv-10.1177_08862605241270047 – Supplemental material for Adverse Childhood Experiences and Police Contact in Canada [file sj-docx-1-jiv-10.1177_08862605241270047.docx]

**Appendix A: Definition and prevalence of specific ACE items**

| **Construct** | **Definition** | **%** |
| --- | --- | --- |
| Emotional Abuse | Did a parent or other adult in your home often insult you or put you down? | 39.3 |
| Threaten Physical Abuse | Did a parent or other adult in your home ever make you afraid that you might be physically hurt? | 33.7 |
| Physical Abuse | Did a parent or other adult in your home ever hit, beat, kick, or seriously hurt you in any way? | 20.4 |
| Sexual Abuse | Were you ever forced to do sexual things you didn’t want to do? | 12.7 |
| Parental Divorce/Separation | Did your parents separate or divorce? | 23.4 |
| Household Domestic Violence | Did your parents or other adults in your home ever physically abuse each other? | 13.0 |
| Household Drinking Problem | Did you ever live with someone who had a drinking problem? | 19.6 |
| Household Substance Abuse | Did you ever live with someone who used illegal drugs or abused prescription drugs? | 12.5 |
| Household Mental Illness | Did you ever live with someone who was depressed or had any other mental health issues? | 55.2 |
| Household Suicide | Did you ever live with someone who attempted suicide? | 10.4 |
| Household Incarceration | Did you ever live with someone who got sent to jail or prison? | 5.1 |
| Emotional Neglect | Did you often feel that no one in your family loved you or thought you were important? | 31.4 |
| Lack of Support | Did you often feel that the members of your family didn’t support one another? | 42.4 |
| Physical Neglect | Did your parents ever fail to meet the basic needs of your family (for example, not providing enough food to eat or clean clothes to wear)? | 6.8 |
| Parental Neglect | Were your parents ever too drunk or high to take you to the doctor when you were sick or hurt? | 2.7 |

Abbreviations: ACE = Adverse Childhood Experience

| **Appendix B: Associations between number of ACEs and any police contact among participants in the Canadian Study of Adolescent Health Behaviors, adjusting for violence and illicit drug use** | | |
| --- | --- | --- |
|  | **OR (95% CI)^a^** | **p** |
| Total number of ACEs |  |  |
| 0 | Ref | Ref |
| 1 | 1.15 (0.65, 2.05) | 0.622 |
| 2 | 0.81 (0.41, 1.59) | 0.546 |
| 3 | 0.96 (0.51, 1.83) | 0.905 |
| 4+ | **1.59 (1.00, 2.52)** | **0.049** |
| Values in **bold** are significant with p < .05.  ^a^ Analyses adjusted for age, race/ethnicity, gender, sexual identity, income, highest level of education completed, violence, and illicit drug use  Abbreviations: ACEs = Adverse Childhood Experience; OR = Odds ratio from logistic regression; CI = Confidence interval | | |

| **Appendix C: Associations between number of ACEs and police stops among participants in the Canadian Study of Adolescent Health Behaviors, adjusting for violence and illicit drug use** | | | | | | | | | | |
| --- | --- | --- | --- | --- | --- | --- | --- | --- | --- | --- |
|  | **Police Stop Intrusion** | | | | **Police Stop Harassment** | | | | | |
|  | **Never stopped vs Stopped w/o intrusion** | | **Never stopped vs Stopped w/ intrusion** | | **Never stopped vs Stopped w/o harassment** | | | **Never stopped vs Stopped w/ harassment** | | |
|  | **RRR (95% CI)^a^** | **p** | **RRR (95% CI)^a^** | **p** | **RRR (95% CI)^a^** | **p** | **RRR (95% CI)^a^** | | **p** |  |
| Total number of ACEs |  |  |  |  |  |  |  | |  |  |
| 0 | Ref | Ref | Ref | Ref | Ref | Ref | Ref | | Ref |  |
| 1 | 1.14 (0.63, 2.85) | 0.449 | 0.97 (0.45, 2.09) | 0.933 | 1.20 (0.64, 2.24) | 0.563 | 1.10 (0.35, 3.49) | | 0.858 |  |
| 2 | 0.77 (0.42, 2.43) | 0.597 | 0.84 (0.36, 1.94) | 0.683 | 0.74 (0.34, 1.61) | 0.445 | 1.21 (0.38, 3.87) | | 0.745 |  |
| 3 | 1.01 (0.42, 2.43) | 0.983 | 0.90 (0.39, 2.08) | 0.812 | 0.84 (0.40, 1.76) | 0.644 | 1.53 (0.50, 7.70) | | 0.455 |  |
| 4+ | 1.33 (0.70, 2.55) | 0.387 | **1.86 (1.04, 3.35)** | **0.038** | 1.06 (0.62, 1.81) | 0.840 | **3.71 (1.58, 8.68)** | | **0.003** |  |
| Values in **bold** are statistically significant at p < 0.05.  ^a^ Analyses adjusted for age, race/ethnicity, gender, sexual identity, highest level of education completed, violence, and illicit drug use  Abbreviations: ACEs = Adverse Childhood Experiences; RRR = Relative risk ratios; CI = Confidence interval | | | | | | | | | | |

| **Appendix D: Adjusted associations between specific ACE type and any police contact among participants in the Canadian Study of Adolescent Health Behaviors** | | | |
| --- | --- | --- | --- |
|  | aOR (95% CI) ^a^ | p |  |
| Specific ACE type |  |  |  |
| Emotional Abuse | 1.04 (0.73, 1.49) | 0.814 |  |
| Threaten Physical Abuse | 1.21 (0.85, 1.73) | 0.281 |  |
| Physical Abuse | 1.33 (0.90, 1.98) | 0.154 |  |
| Sexual Abuse | 1.60 (0.99, 2.59) | 0.054 |  |
| Parental Divorce/Separation | **1.66 (1.12, 2.45)** | **0.011** |  |
| Household Domestic Violence | **2.12 (1.32, 3.39)** | **0.002** |  |
| Household Drinking Problem | 1.24 (0.81, 1.91) | 0.315 |  |
| Household Substance Abuse | **1.80 (1.13, 2.87)** | **0.013** |  |
| Household Mental Illness | **1.57 (1.11, 2.22)** | **0.011** |  |
| Household Suicide | **2.20 (1.33, 3.62)** | **0.002** |  |
| Household Incarceration | **2.25 (1.13, 4.46)** | **0.021** |  |
| Emotional Neglect | 1.16 (0.81, 1.67) | 0.421 |  |
| Lack of Support | 1.33 (0.94, 1.87) | 0.107 |  |
| Physical Neglect | **2.99 (1.63, 5.47)** | **<0.001** |  |
| Parental Neglect | 2.45 (0.91, 6.58) | 0.077 |  |
| Values in **bold** are statistically significant at p < 0.05.  ^a^ Analyses adjusted for age, race/ethnicity, gender, sexual identity, and highest level of education completed  Abbreviations: ACEs = Adverse Childhood Experiences; OR = Odds ratios; CI = Confidence interval | | | |

| **Appendix E: Adjusted associations between specific ACE type and police stops among participants in the Canadian Study of Adolescent Health Behaviors** | | | | | | | | | | | | |
| --- | --- | --- | --- | --- | --- | --- | --- | --- | --- | --- | --- | --- |
|  | Police Stop Intrusion | | | | | | Police Stop Harassment | | | | |  |
| Specific ACE type | Never stopped vs Stopped w/o intrusion | | | Never stopped vs Stopped w/ intrusion | | | Never stopped vs Stopped w/o harassment | | | Never stopped vs Stopped w/ harassment | |  |
|  | RRR (95% CI)^a^ | p | RRR (95% CI)^a^ | | p | RRR (95% CI)^a^ | | p | RRR (95% CI)^a^ | | p |  |
| Emotional Abuse | 0.87 (0.51, 1.48) | 0.604 | 1.24 (0.80, 1.91) | | 0.331 | **0.58 (0.36, 0.96)** | | **0.034** | **2.49 (1.50, 4.14)** | | **<0.001** |  |
| Threaten Physical Abuse | 1.17 (0.71, 1.93) | 0.537 | 1.26 (0.81, 1.95) | | 0.300 | 0.91 (0.57, 1.43) | | 0.669 | **1.89 (1.15, 3.10)** | | **0.012** |  |
| Physical Abuse | 1.17 (0.65, 2.08) | 0.602 | 1.48 (0.91, 2.41) | | 0.115 | 0.88 (0.51, 1.51) | | 0.636 | **2.27 (1.34, 3.86)** | | **0.002** |  |
| Sexual Abuse | 1.02 (0.47, 2.18) | 0.965 | **2.31 (1.31, 4.09)** | | **0.004** | 1.37 (0.72, 2.60) | | 0.337 | **1.95 (1.04, 3.65)** | | **0.038** |  |
| Parental Divorce/Separation | **1.89 (1.11, 3.23)** | **0.020** | 1.48 (0.92, 2.36) | | 0.104 | 1.63 (0.99, 2.65) | | 0.051 | **1.71 (1.01, 2.89)** | | **0.044** |  |
| Household Domestic Violence | 1.66 (0.81, 3.42) | 0.167 | **2.54 (1.49, 4.34)** | | **0.001** | 1.41 (0.75, 2.68) | | 0.286 | **3.63 (2.00, 6.59)** | | **<0.001** |  |
| Household Drinking Problem | 0.96 (0.50, 1.85) | 0.900 | 1.56 (0.96, 2.54) | | 0.074 | 0.98 (0.56, 1.73) | | 0.946 | **1.73 (1.00, 2.98)** | | **0.048** |  |
| Household Substance Abuse | 1.21 (0.59, 2.48) | 0.600 | **2.40 (1.40, 4.13)** | | **0.001** | 1.38 (0.75, 2.53) | | 0.296 | **2.56 (1.42, 4.65)** | | **0.002** |  |
| Household Mental Illness | 1.22 (0.75, 2.00) | 0.422 | **1.97 (1.27, 3.05)** | | **0.002** | 1.11 (0.73, 1.69) | | 0.630 | **3.16 (1.79, 5.58)** | | **<0.001** |  |
| Household Suicide | 1.78 (0.84, 3.74) | 0.129 | **2.58 (1.44, 4.61)** | | **0.001** | **1.99 (1.06, 3.73)** | | **0.032** | **2.58 (1.33, 5.01)** | | **0.005** |  |
| Household Incarceration | 1.43 (0.46, 4.52) | 0.536 | **3.01 (1.43, 6.32)** | | **0.004** | 1.94 (0.82, 4.61) | | 0.132 | **2.77 (1.18, 6.51)** | | **0.019** |  |
| Emotional Neglect | 0.76 (0.44, 1.31) | 0.317 | **1.67 (1.07, 2.61)** | | **0.025** | 0.69 (0.42, 1.14) | | 0.150 | **2.32 (1.40, 3.85)** | | **0.001** |  |
| Lack of Support | 1.23 (0.75, 2.02) | 0.406 | 1.43 (0.93, 2.19) | | 0.103 | 0.91 (0.59, 1.42) | | 0.682 | **2.45 (1.47, 4.08)** | | **0.001** |  |
| Physical Neglect | 1.91 (0.72, 5.05) | 0.190 | **4.13 (2.12, 8.02)** | | **<0.001** | **2.68 (1.26, 5.71)** | | **0.011** | **3.51 (1.64, 7.52)** | | **0.001** |  |
| Parental Neglect | 1.44 (0.24, 8.77) | 0.692 | **3.45 (1.25, 9.54)** | | **0.017** | 1.53 (0.35, 6.74) | | 0.574 | **4.05 (1.34, 12.21)** | | **0.013** |  |
| Values in **bold** are statistically significant at p < 0.05.  ^a^ Analyses adjusted for age, race/ethnicity, gender, sexual identity, and highest level of education completed  Abbreviations: ACEs = Adverse Childhood Experiences; RRR = Relative risk ratios; CI = Confidence interval | | | | | | | | | | | | |

**Appendix F: Associations between the number of ACEs and Any Police Contact among participants in the Canadian Study of Adolescent Health Behaviors (*N* = 940)**

| **Variables** | **aOR (95% CI)^a^** | | **p** | |
| --- | --- | --- | --- | --- |
| Number of ACEs |  |  | |  |
| 0 | Ref | Ref | |  |
| 1 | 1.14 (0.64, 2.01) | 0.656 | |  |
| 2 | 0.86 (0.44, 1.67) | 0.649 | |  |
| 3 | 1.05 (0.56, 1.98) | 0.876 | |  |
| 4 | **1.97 (1.05, 3.71)** | **0.035** | |  |
| 5+ | **1.71 (1.06, 2.76)** | **0.028** | |  |

Values in **bold** are significant with p < .05.

^a^ Analyses adjusted for age, race/ethnicity, gender, sexual identity, income, and highest level of education completed.

*Abbreviations:* ACEs = Adverse Childhood Experiences; aOR = Adjusted Odds Ratio

| **Appendix G. Multinomial logistic regression of relationship between number of ACEs and police stops with intrusion and police stops with Harassment among participants in the Canadian Study of Adolescent Health Behaviors (*N* = 940)** | | | | | | | | | | | |  |
| --- | --- | --- | --- | --- | --- | --- | --- | --- | --- | --- | --- | --- |
|  | **Police Stop Intrusion** | | | | | | **Police Stop Harassment** | | | | | |
|  | **Never stopped vs Stopped w/o intrusion** | | | **Never stopped vs Stopped w/ intrusion** | | | **Never stopped vs Stopped w/o harassment** | | | **Never stopped vs Stopped w/ harassment** | | |
|  | **aOR (95% CI)^a^** | **p** | **aOR (95% CI)^a^** | | **p** | **aOR (95% CI)^a^** | | **p** | **aOR (95% CI)^a^** | | **p** |  |
| Total number of ACEs |  |  |  | |  |  | |  |  | |  |  |
| 0 | Ref | Ref | Ref | | Ref | Ref | | Ref | Ref | | Ref |  |
| 1 | 1.30 (0.62, 2.76) | 0.489 | 0.96 (0.45, 2.07) | | 0.921 | 1.18 (0.63, 2.19) | | 0.604 | 1.10 (0.35, 3.44) | | 0.872 |  |
| 2 | 0.81 (0.31, 2.11) | 0.667 | 0.89 (0.39, 2.04) | | 0.780 | 0.76 (0.35, 1.66) | | 0.488 | 1.34 (0.42, 4.21) | | 0.622 |  |
| 3 | 1.10 (0.46, 2.63) | 0.825 | 1.00 (0.47, 2.27) | | 0.991 | 0.90 (0.43, 1.9) | | 0.772 | 1.74 (0.57, 5.29) | | 0.327 |  |
| 4 | 1.65 (0.67, 4.02) | 0.274 | **2.34 (1.06, 5.18)** | | **0.037** | 1.38 (0.63, 3.05) | | 0.424 | **4.29 (1.55, 11.84)** | | **0.005** |  |
| 5+ | **1.43 (0.71, 2.85)** | **0.314** | **2.01 (1.10, 3.68)** | | **0.023** | 1.08 (0.60, 1.95) | | 0.795 | **4.24 (1.79, 10.01)** | | **0.001** |  |
| Values in **bold** are statistically significant at p < 0.05.  ^a^ Analyses adjusted for age, race/ethnicity, gender, sexual identity, and highest level of education completed.  Abbreviations: ACEs = Adverse Childhood Experiences; aOR = Adjusted Odds Ratio; CI = Confidence interval | | | | | | | | | | | |  |

**Appendix H: Associations between the number of ACEs and any police contact among participants in the Canadian Study of Adolescent Health Behaviors (*N* = 940)**

| **Variables** | **aOR (95% CI)^a^** | | **p** | |
| --- | --- | --- | --- | --- |
| Number of ACEs |  |  | |  |
| 0 | Ref | Ref | |  |
| 1 | 1.14 (0.65, 2.01) | 0.654 | |  |
| 2 | 0.86 (0.44, 1.67) | 0.651 | |  |
| 3 | 1.05 (0.60, 1.99) | 0.872 | |  |
| 4 | **1.97 (1.05, 3.70)** | **0.036** | |  |
| 5 | 1.44 (0.73, 2.81) | 0.293 | |  |
| 6+ | **1.83 (1.10, 3.04)** | **0.002** | |  |

Values in **bold** are significant with p < .05.

^a^ Analyses adjusted for age, race/ethnicity, gender, sexual identity, income, and highest level of education completed.

*Abbreviations:* ACEs = Adverse Childhood Experience; aOR = Adjusted Odds Ratio

| **Appendix I. Multinomial logistic regression of relationship between number of ACEs and police stops with Intrusion and police stops with Harassment among participants in the Canadian Study of Adolescent Health Behaviors (*N* = 940)** | | | | | | | | | | | |  |
| --- | --- | --- | --- | --- | --- | --- | --- | --- | --- | --- | --- | --- |
|  | **Police Stop Intrusion** | | | | | | **Police Stop Harassment** | | | | | |
|  | **Never stopped vs Stopped w/o intrusion** | | | **Never stopped vs Stopped w/ intrusion** | | | **Never stopped vs Stopped w/o harassment** | | | **Never stopped vs Stopped w/ harassment** | | |
|  | **aOR (95% CI)^a^** | **p** | **aOR (95% CI)^a^** | | **p** | **aOR (95% CI)^a^** | | **p** | **aOR (95% CI)^a^** | | **p** | |
| Total number of ACEs |  |  |  | |  |  | |  |  | |  | |
| 0 | Ref | Ref | Ref | | Ref | Ref | | Ref | Ref | | Ref | |
| 1 | 1.30 (0.49, 2.76) | 0.615 | 0.96 (0.45, 2.08) | | 0.925 | 1.12 (0.63, 2.19) | | 0.606 | 1.10 (0.35, 3.45) | | 0.867 | |
| 2 | 0.81 (0.67, 2.11) | 0.312 | 0.89 (0.39, 2.05) | | 0.783 | 0.76 (0.35, 1.70) | | 0.487 | 1.34 (0.43, 4.24) | | 0.614 | |
| 3 | 1.10 (0.83, 2.63) | 0.463 | 1.00 (0.44, 2.28) | | 0.995 | 0.90 (0.43, 1.87) | | 0.769 | 1.76 (0.58, 5.33) | | 0.320 | |
| 4 | 1.65 (0.28, 4.02) | 0.673 | **2.33(1.05, 5.15)** | | **0.038** | 1.38 (0.63, 3.10) | | 0.420 | **4.24 (1.54, 11.71)** | | **0.005** | |
| 5 | 1.41 (0.47, 3.58) | 0.554 | 1.47 (0.61, 3.45) | | 0.406 | 1.31 (0.60, 2.87) | | 0.505 | 2.15 (0.68, 6.73) | | 0.191 | |
| 6+ | 1.43 (0.34, 3.00) | 0.684 | **2.25 (1.20, 4.24)** | | **0.012** | 0.99 (0.52, 1.91) | | 0.981 | **5.23 (2.17, 12.60)** | | **<0.001** | |
| Values in **bold** are statistically significant at p < 0.05.  ^a^ Analyses adjusted for age, race/ethnicity, gender, sexual identity, and highest level of education completed.  Abbreviations: ACEs = Adverse Childhood Experiences; aOR = Adjusted Odds Ratio; CI = Confidence interval | | | | | | | | | | | |  |

**Appendix J: Figure of predicted probabilities of police contact**
